# Dynamic transport and localization of alpha-synuclein in primary hippocampal neurons

**DOI:** 10.1186/1750-1326-5-9

**Published:** 2010-02-09

**Authors:** Mong-Lin Yang, Linda Hasadsri, Wendy S Woods, Julia M George

**Affiliations:** 1 Department of Cell and Developmental Biology, University of Illinois at Urbana-Champaign, Urbana, IL, USA; 2 Medical Scholars Program, University of Illinois at Urbana-Champaign, Urbana, IL, USA; 3 Department of Molecular and Integrative Physiology, University of Illinois at Urbana-Champaign, Urbana, IL, USA

## Abstract

**Background:**

Alpha-synuclein is a presynaptic protein with a proposed role in neurotransmission and dopamine homeostasis. Abnormal accumulation of α-synuclein aggregates in dopaminergic neurons of the substantia nigra is diagnostic of sporadic Parkinson's disease, and mutations in the protein are linked to early onset forms of the disease. The folded conformation of the protein varies depending upon its environment and other factors that are poorly understood. When bound to phospholipid membranes, α-synuclein adopts a helical conformation that mediates specific interactions with other proteins.

**Results:**

To investigate the role of the helical domain in transport and localization of α-synuclein, eGFP-tagged constructs were transfected into rat primary hippocampal neurons at 7 DIV. A series of constructs were analyzed in which each individual exon was deleted, for comparison to previous studies of lipid affinity and α-helix content. A53T and A30P substitutions, representing Parkinson's disease-associated variants, were analyzed as well. Single exon deletions within the lipid-binding N-terminal domain of α-synuclein (exons 2, 3, and 4) partially disrupted its presynaptic localization at 17-21 DIV, resulting in increased diffuse labeling of axons. Similar results were obtained for A30P, which exhibits decreased lipid binding, but not A53T. To examine whether differences in presynaptic enrichment were related to deficiencies in transport velocity, transport was visualized via live cell microscopy. Tagged α-synuclein migrated at a rate of 1.85 ± 0.09 μm/s, consistent with previous reports, and single exon deletion mutants migrated at similar rates, as did A30P. Deletion of the entire N-terminal lipid-binding domain (Δ234GFP) did not significantly alter rates of particle movement, but decreased the number of moving particles. Only the A53TGFP mutant exhibited a significant decrease in transport velocity as compared to ASGFP.

**Conclusions:**

These results support the hypothesis that presynaptic localization involves a mechanism that requires helical conformation and lipid binding. Conversely, the rate of axonal transport is not determined by lipid affinity and is not sufficient to account for differences in presynaptic localization of α-synuclein-eGFP variants.

## Background

Alpha-synuclein (α-syn) is a 140-amino acid protein enriched at presynaptic terminals of the vertebrate central nervous system [1], although significant localization to other subcellular compartments (nucleus[2,3], endoplasmic reticulum[4] and other cell types, including various glia [5,6]) has also been observed. Its physiological function is not well understood, but it has been implicated in the regulation of activity-dependent plasticity [7,8], dopamine biosynthesis [9], and membrane dynamics at the presynaptic terminal [10,11]. The protein is intrinsically unstructured [12], but adopts an α-helical secondary structure when bound to phospholipid membranes [13]; this induced structure mediates specific interactions with other proteins [14]. An alternative β-sheet conformation of α-syn is associated with a number of neurodegenerative disorders, most prominently Parkinson's disease (PD). Misfolded α-syn is the main fibrillar component of Lewy bodies and Lewy neurites [15,16], intraneuronal inclusions that are typical of this disorder. Mutations in α-syn are associated with rare familial forms of Parkinson's disease. These mutations include point mutations [17-19] and duplications or triplications of the gene SNCA [20-22], which encodes α-syn protein.

The basis of α-syn localization to the presynapse is unclear. Unlike many classical presynaptic proteins, α-syn is not an integral membrane protein of synaptic vesicles [1,23,24], although it may associate transiently with these structures [25,26]. Furthermore, its localization to the synapse in cultured neurons occurs several days after functional synapses first form, suggesting that α-syn is not essential for neurotransmitter release [27]. Recent studies have demonstrated that α-syn undergoes axonal transport via slow component b (SCb), a process requiring an intact microtubule cytoskeleton and driven by the motor proteins kinesin and dynein [28,29]. Consistent with this model, we have found conformation-dependent interactions between helical (lipid-bound) α-syn and the motor proteins kinesin light chain 1S (KLC1S) and the heavy chain of cytoplasmic dynein (DHC1) [14].

In the present study, we sought to determine whether membrane binding, and the resultant stabilization of helical protein conformation, is essential for either transport or localization of α-syn to the presynaptic terminal, using eGFP-tagged α-syn constructs expressed in rat hippocampal primary neuronal cultures. A similar eGFP-tagging strategy has been used successfully in studies of another small presynaptic protein, synapsin, which was found to localize normally even in the presence of the large eGFP tag [30]. We systematically deleted each coding exon, to facilitate correlation of transport and localization behavior with our previous studies [13,31] of lipid affinity and α-helix content. We also analyzed the PD-associated mutants A53T and A30P. We found that deletions of single exons within the lipid-binding N-terminal domain of α-syn (exons 2, 3, and 4) or the PD-association mutation A30P partially disrupted the localization of α-syn at presynaptic terminals, resulting in increased diffuse labeling of axons. A53T exhibited normal presynaptic enrichment, but its transport velocity was decreased relative to the other variants. Finally, we observed that deletion of lipid-binding exons 2-4 resulted in a decreased number of motile, axonal α-syn-containing particles without affecting instantaneous velocity of those particles.

## Results

**Figure 1:**
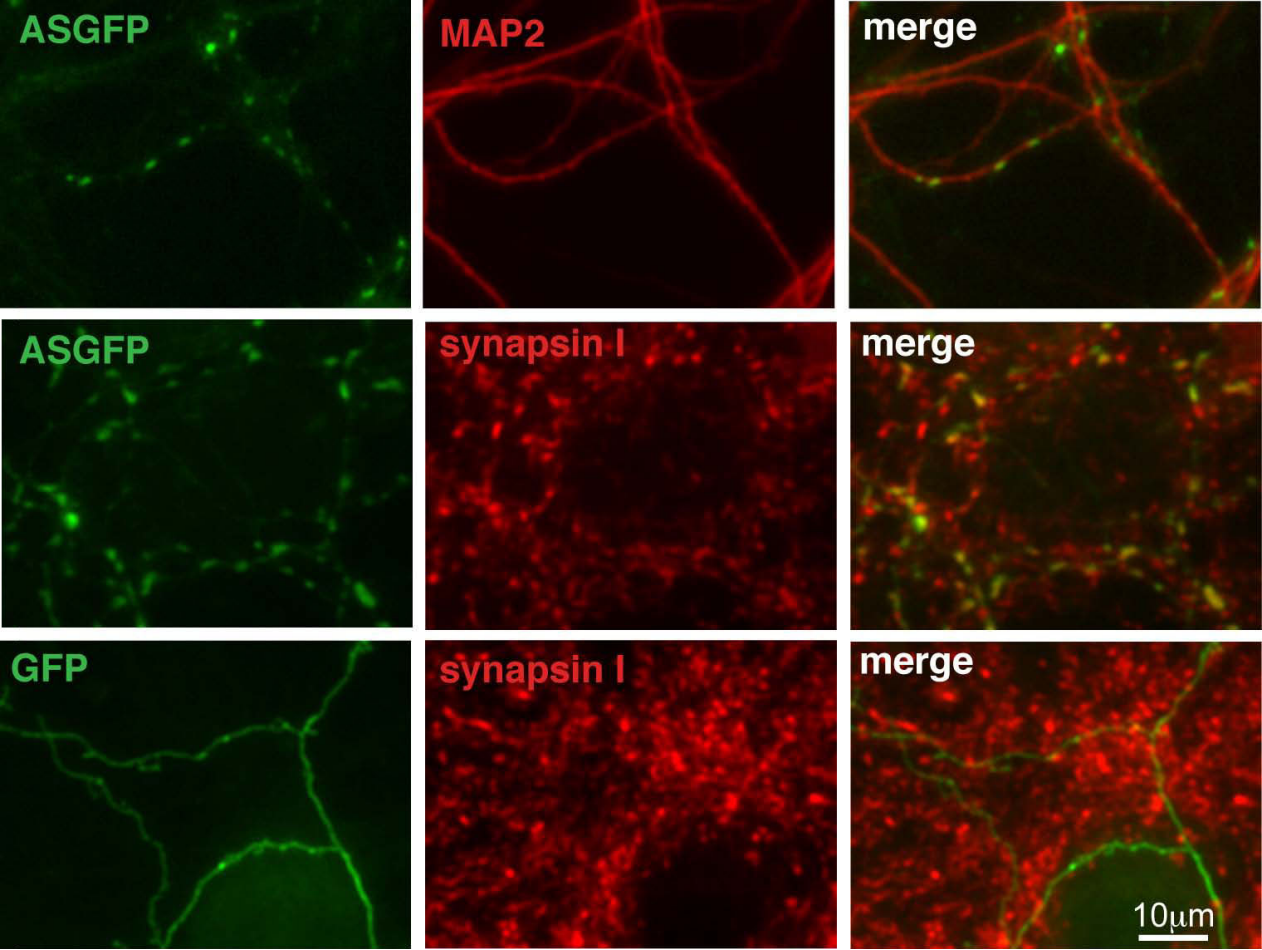
**ASGFP localizes to the presynaptic terminal by 17DIV**. Primary rat E18 hippocampal neurons were transfected with either ASGFP (top and middle rows, green) or eGFP (bottom row, green). After 17 days *in vitro *(DIV), cells were fixed and immunostained for MAP2 (top row, red), or synapsin I (middle and bottom rows, red), and visualized for immunofluorescence. Scale bar = 10 μm.

To facilitate analysis of the protein domains required for targeting of α-syn to presynaptic terminals, we expressed a fusion construct encoding full-length human α-syn with a C-terminal eGFP tag (ASGFP). As observed previously for wild-type α-syn [27], ASGFP stably localizes to presynaptic terminals of primary hippocampal neurons by approximately 14-21 days *in vitro *(DIV). In Fig. 1, punctate α-syn staining is observed adjacent to MAP2 positive dendrites. The α-syn staining overlaps with staining for the presynaptic protein synapsin I. In contrast, eGFP alone is distributed diffusely within neuronal processes, and is not concentrated at synapses.

**Figure 2:**
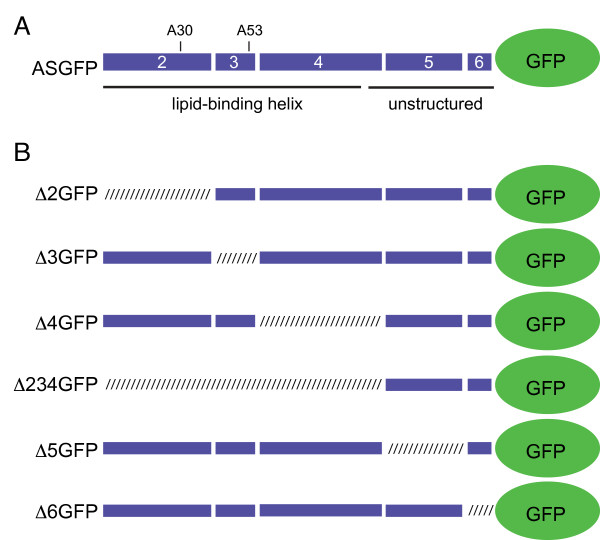
**Schematic representation of α-synuclein/eGFP constructs**. (A) Schematic of wild-type α-syn with C-terminal eGFP fusion; coding exons are depicted as blue boxes numbered 2 to 6. Positions of two PD-associated mutations (A30 and A53) are indicated, and black lines represent the predicted secondary structure in the presence of lipid. (B) Exon deletion mutants; crosshatched boxes represent deleted regions.

Having thus established that tagged ASGFP localizes similarly to untagged α-syn, we next analyzed a series of deletion constructs corresponding to the natural exons in the SNCA gene (Fig. 2). Five exons (numbered 2 through 6) form the longest splice variant of α-syn (α-syn 1-140). Exons 2 through 4 (Fig. 2) encode an amphipathic α-helical domain shown previously to mediate reversible interactions with lipid [31]. We tested deletions of each of the exons individually (Δ2GFP, Δ3GFP, etc.), and of the entire lipid-binding domain (Δ234GFP). We also tested eGFP-tagged constructs corresponding to two known Parkinson's disease-associated mutations, A30PGFP and A53TGFP.

**Figure 3:**
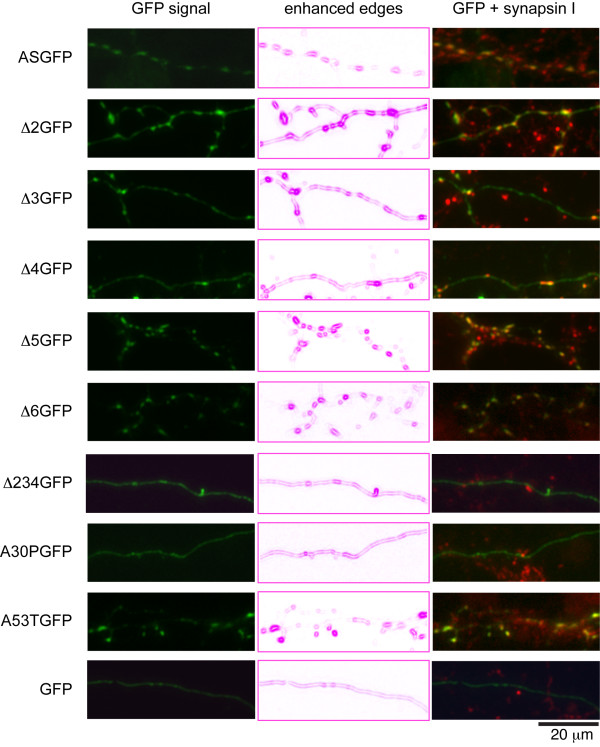
**Localization pattern of exon-deletion mutants**. Primary rat E18 hippocampal neurons were transfected with α-syn variant constructs. After 17 DIV, cells were fixed and immunostained for synapsin I. The left column shows the eGFP signal from the various α-syn constructs (green). The middle employs the "find edges" filter in Adobe Photoshop, which represents color transitions as lines. The right column shows a merge of the eGFP signal from the various α-syn constructs (green) with the synapsin I staining (red).

**Figure 4:**
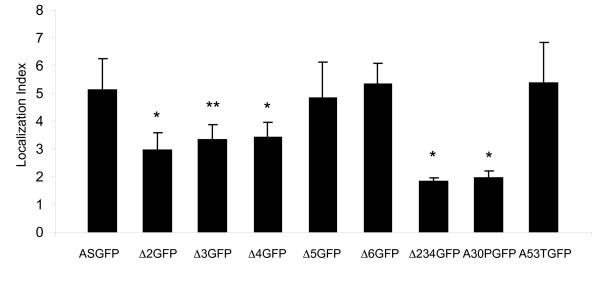
**Quantification of localization**. Bar graph showing the localization index of each construct expressed in neurons. The localization index is a ratio of average signal intensity from boutons versus average signal intensity from axonal processes. The mean localization index of each mutant (n = 15 cells) was compared to ASGFP using pairwise t-test with Bonferroni correction for repeated measures (*p < 0.0001, **p = 0.0002).

Each construct was transfected into primary hippocampal neurons and visualized at 17-21 DIV. ASGFP was observed to localize at discrete punctae along axonal processes, with minimal signal detected in intervening axonal segments (Fig. 3). These punctae presumably represent presynaptic boutons, as most were immunopositive for synapsin I. In contrast to ASGFP, mutants with partial deletions of the N-terminal helical domain (Δ2GFP, Δ3GFP, and Δ4GFP) were localized to axonal punctae, but also maintained residual diffuse staining along the axon. To quantitate this effect, we calculated the ratio of the fluorescence intensity at presynaptic terminals to the signal along the axon, and compared the resulting localization index for all the mutants (Fig. 4). We found that the localization index was significantly reduced for Δ2GFP, Δ3GFP, and Δ4GFP, and that the effect was even more pronounced for Δ234GFP. Δ5GFP and Δ6GFP, each with partial deletions of the unstructured C-terminus, were indistinguishable in localization from the wild-type construct. For the PD mutants, we found that A30PGFP exhibited a significant attenuation of its localization index, while A53TGFP was unimpaired in its localization.

**Figure 5:**
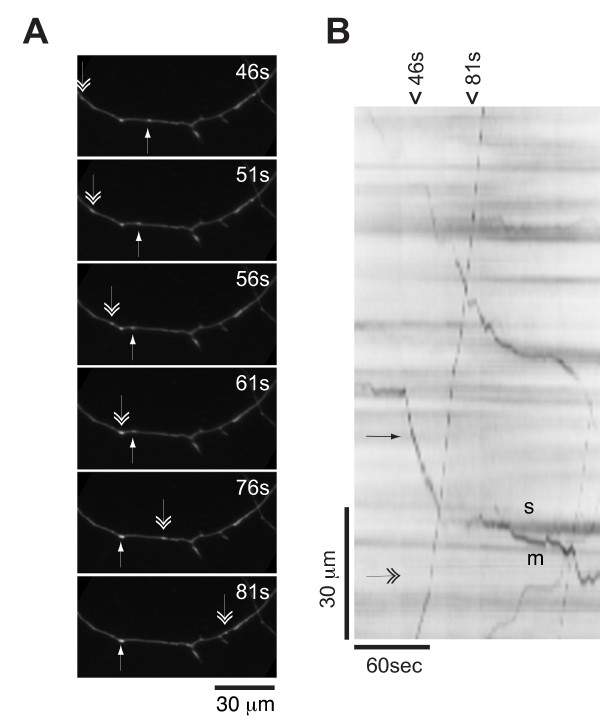
**ASGFP transport behavior**. (A) Time lapse fluorescent images of ASGFP transport particles in rat primary hippocampal neurons visualized at 10 DIV. The single-headed arrow indicates an ASGFP structure that moves along the axon, while the double-headed arrow indicates an ASGFP structure moving in the opposite direction. (B) Kymograph of the full time lapse series, with the time window depicted in A noted on top (46-81s). The particles are labeled as in panel A. One of the particles (double-headed arrow) is seen to move continuously in one direction, while other particle (single-headed arrow) moves in the opposite direction, pauses, and then bifurcates into stationary (s) and motile (m) particles.

We reasoned that a failure to concentrate at presynaptic sites could result from deficits in transport to synapses, impaired retention at synapses, or both. With respect to the former, active axonal transport of α-syn has been documented previously (see Discussion). We measured migration of ASGFP-positive particles along neuritic processes at 10-14 DIV, prior to establishment of a stable presynaptic localization. These particles were observed by time-lapse microscopy to move bidirectionally within the same neuritic process (Additional file 1), at times slowing and appearing to interact with more stationary particles. In Fig. 5A, images from a time-lapse series illustrate the behavior of two particles moving in opposite directions along the same neurite (double and single arrowheads, respectively).

For analysis of particle velocity, time-lapse series were rendered as kymographs (see Methods). In these kymograph plots, velocity is represented by the slope of each line (y length over x time), and a pause in movement is represented by a horizontal segment. When the series from Fig. 5A is rendered as a kymograph (Fig. 5B), it is evident that the ASGFP particles move with different velocities. One particle (double arrowhead) moves rapidly through the length of the frame, while another particle (single arrowhead) is initially stationary, then moves a short distance and pauses before splitting into two particles, one stationary (labeled "s") and one continuing in the original direction of movement (labeled "m") (Fig. 5B).

**Figure 6:**
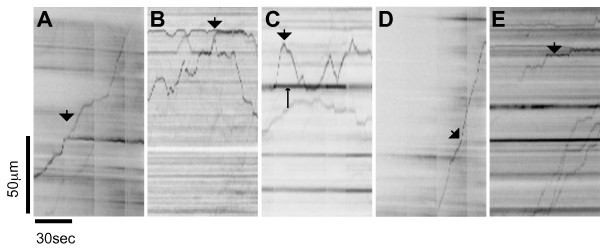
**Examples of ASGFP transport behavior**. Kymographs illustrating examples of ASGFP transport behavior. (A) One particle is clearly seen to bifurcate into two distinct particles (bold arrow). (B) Three starting particles briefly fuse to become one particle (bold arrow) before bifurcating again into two particles. (C) One particle (bold arrow) moves bidirectionally in and out of a stationary accumulation of ASGFP (fine arrow) (D) One particle moves without stopping or changing direction (bold arrow) (E) One particle gradually emerges from the background (bold arrow). (vertical scale bar = 50 μm, horizontal scale bar = 30s).

Other examples of ASGFP behavior are illustrated in Fig. 6. In Fig. 6A, one particle is clearly seen to bifurcate into two distinct particles; of these, one continues its motion down the axon, while the other remains stationary throughout the duration of measurement. In Fig. 6B, three starting particles interact in a complex fashion, moving bidirectionally, fusing, and then splitting again. Fig. 6C demonstrates how some particles move bidirectionally in and out of a stationary accumulation of α-syn, while Fig. 6D represents how some particles are seen to travel in one direction without stopping during the period of imaging. Last, in Fig. 6E, a distinct particle slowly emerges, apparently due to the accumulation of diffuse ASGFP signal.

**Figure 7:**
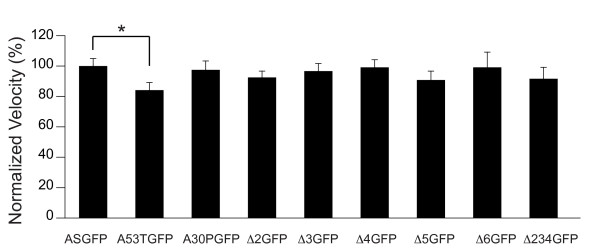
**Velocity distribution for ASGFP and mutants**. Velocities during periods of movement were calculated from kymographs, and mean ± SEM plotted as a percentage of ASGFP velocity. A53TGFP velocity was decreased relative to ASGFP (*p = 0.0033, pairwise t-test with Bonferroni correction; n>50 movies per group).

Velocities were calculated for migrating particles of mutant and wild-type ASGFP. Only objects with continuous net movements of at least 10 μm were analyzed and only during periods of motion. In each case a broad distribution of velocities was measured (Fig. 7 and Table 1). When these data were compared by pairwise t-test, a slight decrement in mean particle velocity was measured only for A53T.

**Table 1:** Mean velocities of ASGFP and mutants.

protein variant	mean velocity (μm/sec) ± SEM	p value
ASGFP	1.85 ± .09	

A53TGFP	1.56 ± .08	0.0033 *

A30PGFP	1.80 ± .11	0.3409

Δ2GFP	1.71 ± .08	0.0111

Δ3GFP	1.79 ± .09	0.8218

Δ4GFP	1.84 ± .08	0.3064

Δ5GFP	1.68 ± .10	0.1065

Δ6GFP	1.83 ± .18	0.6275

^†^ASGFP	2.11 ± .09	

^†^Δ234GFP	1.93 ± .14	0.0922

Data were normalized by log transformation, and a pairwise t-test was performed with Bonferroni correction for multiple comparisons. ^†^Δ234GFP was compared to ASGFP in an independent experiment, and p values were determined within experiment.

*significantly different from ASGFP.

**Figure 8:**
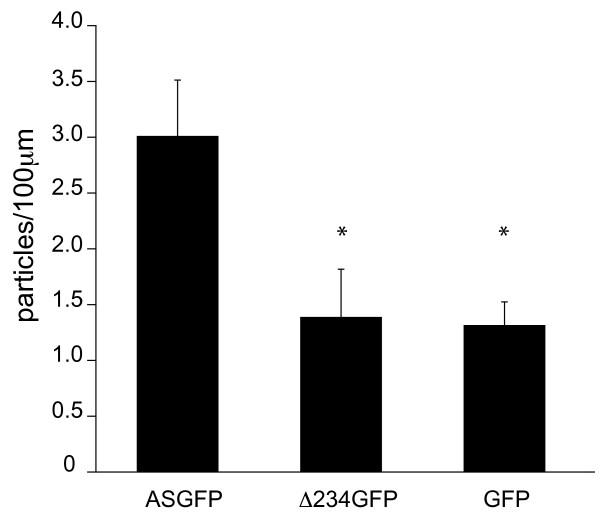
**Quantitation of motile particles**. Primary hippocampal neurons were transfected at 7 DIV. Kymographs were obtained from the proximal axon segment at 10-14 DIV, and particles displaying >10 μm continuous movement were counted and normalized to the length of the axon segment. (*p < 0.02 for the comparison with ASGFP, one-way ANOVA with post-hoc Tukey test, n ≥ 9 cells per group)

However, we measured fewer moving particles overall for the Δ234GFP variant (Fig. 8). Primary hippocampal neurons were transfected with ASGFP, Δ234GFP, or eGFP as a control, and the number of moving particles in the proximal axon segment of each cell was normalized to its length. Cells transfected with ASGFP contained approximately twice the number of motile particles as cells transfected with Δ234GFP, which were comparable to background (eGFP alone). These results suggest that the N-terminal domain of α-syn may mediate its association with transport particles.

## Discussion

Intracellular inclusions of α-syn are characteristic of a variety of neurodegenerative disorders, collectively termed "synucleinopathies", of which Parkinson's disease is most prominent [32]. In PD, α-syn inclusions can be either perinuclear (Lewy bodies) or axonal (Lewy neurites). In healthy brain, the distribution of α-syn is primarily presynaptic, although nuclear localization has also been noted. In primary hippocampal neurons, α-syn protein is initially expressed in the cell soma, and becomes progressively enriched at presynaptic terminals over several weeks. PD thus involves a mislocalization of α-syn, and may reflect a failure of the normal processes of axonal transport and localization of the protein.

In this study, axonal transport and presynaptic localization of α-syn were dissected via systematic deletion of each of the 5 coding exons of α-syn. Prior studies in our laboratory have shown that exons 2, 3, and 4, which span residues 1-41, 42-56, and 57-102, respectively, adopt an α-helical conformation in the presence of lipid, and contribute to cooperative interactions with phospholipid membranes [31]. Exons 5 and 6 encode a domain without defined secondary structure, and do not contribute to membrane binding [33]. Here we show that eGFP-tagged mutants of α-syn, each lacking one of the first three coding exons (Δ2GFP, Δ3GFP, and Δ4GFP), do localize to presynaptic terminals, but with a lower degree of presynaptic versus axonal enrichment when compared to the full-length α-syn construct (ASGFP). A construct in which all three lipid-binding exons were deleted (Δ234GFP) showed much greater impairment, while C-terminal mutants (Δ5GFP and Δ6GFP) were indistinguishable from full-length ASGFP. These results demonstrate a positive correlation between membrane binding and presynaptic enrichment of α-syn deletion mutants.

We also measured the presynaptic enrichment of the PD mutants A53TGFP and A30PGFP. Prior studies have indicated that A53T binds membranes with similar or even greater affinity than wild-type α-syn [31]. In contrast, A30P has a proline residue in exon 2, which tends to disrupt the N-terminal lipid-binding α-helix [34], decreasing its affinity for lipid membranes [31]. We found that A53T was enriched at presynaptic terminals of primary hippocampal neurons, while A30P was much less enriched, relative to wild-type ASGFP. This too is consistent with a role for lipid-binding in normal presynaptic localization.

Using viral expression of α-syn GFP constructs, Specht et al. have previously reported that presynaptic localization of α-syn depends on an intact N-terminus [35]. In contrast to our results, they found that deletion of residues 1-19 was sufficient to completely inhibit α-syn localization to the presynapse, while we found that an even more extensive deletion (Δ2GFP, lacking residues 1-41) only partially inhibited localization. The time courses of our respective experiments were quite different: they measured localization of α-syn after just 5 hours of viral infection, while we made our measurements after ~3-7 days of transfection. It is possible that the Specht et al. results reflect differences in initial transport to the presynapse that are not obvious after longer periods of equilibration. Indeed, Saha et al. [36] observed decreased transport velocity of A53T and A30P α-syn after 3-4 hours of transfection that were not measurable at 5-6 hours.

The use of an exon-based deletion strategy yields potential insight into the function of natural splice variants of α-syn. For example, the splice variant SNCA 126, which lacks exon 3 (similar to Δ3GFP), is expressed at relatively lower levels in the frontal cortex of demented patients [37]. Given our observation that Δ3GFP is localized less efficiently to presynaptic terminals than the full-length isoform, we speculate that a difference in protein localization may underlie the association between SNCA 126 expression and dementia.

Our conclusion that membrane binding is required for presynaptic localization is consistent with the observations of Fortin et al., who report that drugs known to alter cholesterol and sphingolipid synthesis also disrupt the presynaptic localization of α-syn [38]. Additional studies by the same investigators indicate that presynaptic localization requires membrane microdomains enriched for phosphatidylserine headgroups and specific polyunsaturated side chains [39]. They also note a specific decrease in lipid affinity and presynaptic localization by N-terminally eGFP-tagged A30P [26,38]. Taken together, the available evidence suggests that the selective concentration of α-syn at presynaptic terminals is dependent, at least in part, on its affinity for presynaptic membranes. This affinity is sensitive to perturbations of the N-terminal helical domain of α-syn (Δ2GFP, Δ3GFP, Δ4GFP, A30PGFP), and depends on the presence of specific lipid microdomains within the presynaptic terminal.

Another factor potentially influencing the presynaptic localization of α-syn is its trafficking along the axon. Numerous prior studies indicate that α-syn is carried via slow axonal transport, particularly in slow component b (SCb) [28,29,40-42]. Initial experiments measured bulk transport of labeled α-syn over hours to days, reflecting net movement of the population of α-syn molecules *in vivo*. Jensen et al. [40] measured transport of ^35^S-methionine labeled α-syn in the rat visual system, and concluded that the bulk of α-syn was transported in SCb. Similarly, Li et al. [41] measured transport of α-syn in peripheral nerves of A53T and A30P transgenic mice, and reported that all forms of α-syn (mutant and wild-type) were transported in the slow component. Subsequent studies extended these results to cell culture models, allowing for more acute visualization of the transport process. Utton et al. [28] performed time-lapse imaging of α-syn-eGFP in cortical neurons, and measured behavior consistent with SCb: periods of fast transport (average velocity 161 mm/day, or 1.86 μm/s) interspersed with pauses, and frequent changes in the direction of movement.

We observe similar behavior for eGFP-tagged wild-type α-syn (ASGFP) in primary rat hippocampal neurons analyzed at 10-14 DIV. In our experiments, ASGFP exhibited a mean velocity during periods of movement of ~1.85 μm/s, or 155 mm/day. This rate, if sustained, would be consistent with fast axonal transport. Only rarely, however, did ASGFP transport particles move continuously during the period of observation; most particles exhibited bidirectional movement punctuated by frequent pauses. Such behavior is typical of SCb cargoes, which have been shown to utilize the fast transport machinery yet achieve a lower net transport velocity as a consequence of a decreased duty ratio [43].

We applied the exon deletion strategy to assess the structural requirements for axonal transport of α-syn. None of the individual exon deletions altered the mean transport velocity during periods of movement, nor did the more extensive deletion Δ234GFP. Likewise, transport of A30PGFP was statistically identical to ASGFP. Only A53TGFP showed a slight decrement in mean transport velocity. These results indicate that the decreased presynaptic localization observed for Δ2GFP, Δ3GFP, Δ4GFP, Δ234GFP, and A30PGFP is not simply attributable to a failure of axonal transport, and suggest that differences in localization may be mediated by a specific lipid-dependent mechanism for retention of alpha-synuclein at the presynaptic terminal, consistent with previous reports[26,38]. However, we were unable to distinguish anterograde and retrograde transport in our assays, so we cannot exclude the possibility that the variants we tested might have specific effects on these different components that are lost when the data are aggregated.

However, association with the transport machinery seems to partially depend upon the N-terminus, as complete deletion of this domain (Δ234GFP) resulted in a decrease in the number of transport particles per unit axon length, similar to eGFP alone. This implies that the formation of transport particles may depend upon the helical N-terminus, while the migration rate of formed particles does not. Consistent with a role for the N-terminal helical domain in axonal transport, we recently reported a number of conformation-dependent protein binding partners of α-syn, including the microtubule motors kinesin light chain 1S (KLC1S) and dynein heavy chain I (DHC1) [14]. These partner proteins were identified in a phage display screen using vesicle-bound α-syn as "bait". Since vesicle binding induces a conformational change in the N-terminal domain encoded by exons 2-4, these conformation-dependent interactions are likely mediated by the N-terminus, as was confirmed through a detailed structural characterization of the interaction between α-syn and endosulfine-alpha [14,44]. Our observation of a lipid-dependent interaction between α-syn and KLC1S might account for the inability of Utton et al. [28] to detect a direct interaction between purified α-syn and kinesin, despite abundant evidence for anterograde transport of α-syn. Recently Roy et al. have reported co-transport of α-syn, synapsin 1, and GAPDH in SCb, and have proposed that these proteins, and perhaps others, could form a multiprotein complex [42]. We also identified synapsin 1a as a conformation-dependent binding partner of α-syn [14]. It is thus intriguing to speculate that α-syn might function as a scaffold mediating microtubule-dependent axonal transport of a macromolecular complex containing lipid (vesicular membranes or other lipid-containing particles) and specific proteins.

## Conclusions

Presynaptic localization of eGFP-tagged α-syn and mutant constructs correlated well with previous measurements of lipid affinity and α-helix content. Deletion of residues encoded by exons 2, 3, or 4 decreased the relative enrichment of α-syn at presynaptic sites at 17-21 DIV, as did the A30P substitution. Presynaptic enrichment was not correlated with transport velocity, as only A53T, which binds lipid with a similar affinity as wild-type protein, showed a significant deficit in axonal transport velocity. However, variations in presynaptic enrichment could relate to other aspects of axonal transport, including propensity to associate with transport particles, or retention at specific sites following transport.

## Methods

### Cell culture and transfection

E18 rat hippocampi (BrainBits Inc., Springfield, IL) were dispersed and seeded at 1.6 × 10^4 ^cells per cm^2 ^onto poly-D-lysine coated glass coverslip culture plates (MatTek, Ashland, MA). Cultures were grown in Neurobasal/B27 (Invitrogen, Carlsbad, CA) supplemented with 0.5 mM glutamine and 25 μM glutamate at 37°C, 5% CO_2_. Half the volume of medium (without glutamate) was replaced every 3-4 days.

Neurons were transfected at 7 DIV using Lipofectamine 2000 (Invitrogen). Transfection was carried out using 1 μg DNA plus 0.5 μl Lipofectamine 2000 per well as per manufacturer instructions. Neurons were incubated with the transfection mixture for 10 minutes, and then switched immediately to Neurobasal/B27 for one hour. The cells were washed three times with PBS and subsequently cultured in fresh medium. Transfection efficiencies of 1-3% were routinely obtained for all constructs.

All GFP-tagged and mutant constructs of α-syn were previously generated by Dr. Richard Perrin using a pEGFP-N3 vector from Clontech (Mountain View, CA).

### Immunofluorescence

Neurons were fixed at 17-21 DIV using 4% paraformaldehyde for 20 minutes. After washing three times with PBS, the cells were then permeabilized for 15 minutes using 0.25% Triton X-100 (Sigma-Aldrich, St. Louis, MO). Following three more washes with PBS, the samples were blocked in 10% BSA for 1 hour at 37°C, incubated with primary antibodies (Mouse anti-Synuclein (H3c), our own lab; Chicken anti-MAP2, Covance; Rabbit anti-Synapsin I, Zymed) in 2% BSA for 1 hour at 37°C, washed three more times in PBS, then incubated with fluorescently labeled secondary antibody (Alexa Fluor 488 goat anti-mouse, Alexa Fluor 546 goat anti-rabbit and Alexa Fluor 633 goat anti-chicken, Invitrogen) as before. After three more washes with PBS the samples were mounted onto microscope slides (Fisher) using 2% PVA-DABCO (Sigma-Aldrich) and dried overnight at room temperature. The cells were then visualized on a Leica DM IRE2 fluorescence microscope (Leica Microsystems, Bannockburn, IL). Images were acquired using OpenLab Software (Improvision Inc., Lexington, MA). For quantification of localization, all images were acquired at 40× magnification. 15 images were taken for each α-syn variant. Within each image, there were multiple processes and each was traced using the Neuro J program of ImageJ to identify regions of interest (ROI) of at least 100 μm per sample. ROIs were finally exported as numerical profiles representing the pixel intensity measured along that axonal stretch. The readouts were then used to generate average pixel intensity for the synapse versus axon. Statistic analysis was done by pairwise t-test with Bonferroni correction using SAS software.

### Live cell imaging

Live fluorescence microscopy was performed on neurons at 10 DIV. Cells were first rinsed with Hibernate E (BrainBits, Springfield, IL) as Hibernate E allows maintenance of neurons in ambient carbon dioxide. Hibernate E containing 2% B27 and 2 mM glutamine was subsequently added and used for the remaining imaging period. Imaging was conducted within a Leica ML incubator housing a Leica DM IRE2 fluorescent inverted microscope. Cells were maintained at 37°C using a Tempcontrol 37-2 digital controller (Pecon GmbH, Germany). Images were acquired at 40× magnification via OpenLab software and binned (2 × 2) with an exposure time of 400 ms.

### Kymograph analysis

OpenLab was used for acquiring images at a resolution of 768 × 1024 pixels. Movie segments of 100 frames (~3 minutes imaging time) containing ~100 μm of axonal processes with moving particles were extracted from the original images. Maximum Z-projection through the time series was applied for clearer identification of ROI using the Neuron J module of ImageJ (NIH). After ROIs were identified, kymographs were generated using the ImageJ plugin "Multiple Kymograph" [45] to facilitate representation of moving structures in two dimensions. Intensity values along the ROI are calculated and plotted with respect to time and position along the ROI. On the x-axis, each pixel represents ~1.7s, while on the y-axis, each pixel represents ~0.526 μm. Moving structures meeting predefined intensity and contrast criteria appear as contrast edges, the slope of which can be used to extrapolate velocity. For velocity measurements, only objects with continuous net movements of at least 10 μm were analyzed and only during periods of motion. Statistical comparisons of the velocity distributions were performed using SAS software. The data were first normalized by log transformation, and then a pairwise t-test was performed with Bonferroni correction for repeated measures.

## Competing interests

The authors declare that they have no competing interests.

## Authors' contributions

MLY designed and performed the experiments, collected and analyzed the data, and participated in preparation of the manuscript. LH assisted in experimental design, data analysis, and preparation of the manuscript. WSW designed and performed experiments, and participated in data analysis and preparation of the manuscript. JG contributed to experimental design and data interpretation, statistical analyses, and preparation of the manuscript. All authors read and approved the final manuscript.

## Supplementary Material

Additional file 1 **Bidirectional movement of ASGFP particles**. Cultured rat hippocampal neurons were transfected with ASGFP at 7DIV, with live images acquired at 10 DIV. Arrows (appearing at 32s and 40s, respectively) indicate distinct particles traveling in opposite directions. The time stamp at the top right is shown in min:sec:ms and scale bar = 100 μm. The image series is acquired at 1.7s/frame and played back at 0.2s/frame. Click here for file
